# Assessing the quality of water used for vegetable irrigation in Tamale Metropolis, Ghana

**DOI:** 10.1038/s41598-021-84617-8

**Published:** 2021-03-05

**Authors:** Clement Kamil Abdallah, Khaldoon A. Mourad

**Affiliations:** 1 Institute of Water and Energy Sciences (Incl. Climate Change), Pan African University, 13000 Tlemcen, Algeria; 2 Lund University, 22100 Lund, Sweden; 3 The Centre for Sustainable Visions, 22643 Lund, Sweden; 4 Ghana Integrated Water, Sanitation and Hygiene, World Vision, Tamale, Ghana

**Keywords:** Environmental sciences, Environmental chemistry

## Abstract

This paper assesses water quality that is used for vegetable irrigation in Tamale Metropolis, Ghana. A mixed-method of research design was employed in this study to collect and analyze the data based on survey instruments. The paper found that *Escherichia coli* (*E. coli*) that is usually used as an indicator of water contamination and heavy metals exist in all taken water samples. The mean concentrations of nutrients such as ammonia, nitrate, nitrite and phosphate were recorded as 0.022 mg/l to 5.98 mg/l for ammonia, 1.06 mg/l to 7.52 mg/l for nitrate, 0.031 mg/l to 0.056 mg/l for nitrate and 0.037 mg/l to 0.069 mg/l for phosphate. *E. coli* and Total Coliforms levels for Sanghani, Kamina and Waterworks from the laboratory analysis were recorded as 3.2 × 10^3^ CFU 100 m/l and 5.5 × 10^2^ CFU 100 m/l, 4.0 × 10^3^ CFU 100 m/l and 1 × 10^2^ CFU 100 m/l, and 2.1 × 10^3^ CFU 100 m/l and 4.6 × 10^2^ CFU 100 m/l respectively. To conclude, based on the measured parameters, water used for irrigation in the Tamale Metropolitan is polluted and may cause potential health risks. Therefore, farmers, traders and consumers need to apply further safety measures to make the vegetables safe.

## Introduction

### Background

The use of wastewater for irrigation is much more commonplace than generally believed. This is because the vast majority of the developing world highly depends on it to augment declining water resource for food production^1^. It is estimated that about 20 million hectare of land is irrigated worldwide using wastewater^2^, other studies estimate wastewater irrigated land as the same size as Germany which stands at about 29.3 million hectares^3^. Due to the water shortage, the absence of laws and authorized body, people use wastewater or low water quality for irrigation^4^. Moreover, the lack of proper sanitation and wastewater treatment systems in Africa increased the chances of water, sanitation, and hygiene (WASH) related diseases^5^ and made wastewater as an open source that is discharged to water bodies^6^.

The use of wastewater for irrigation is not limited to the developing world. Industrialized countries in Europe and America also make use of wastewater for agriculture^7^. Wastewater use in the developing world varies with a significant gap to that of the developed world. In developing countries, wastewater is often characterized as untreated, very low quality and poses a high risk in terms of health to consumers and farmers^8^. However, the developed world with advanced technology is able to treat wastewater for irrigation and other purposes^9^. The use of wastewater for irrigation is increasing in exponentially in Africa and Asia due to population growth, high demand for perishable goods and water scarcity coupled with poverty^10^.

In 2006, the World Health Organization (WHO) published guidelines for the safe use of excreta and greywater use in agriculture^11^ and guidelines for wastewater use in agriculture^12^, which were considered straightforward to be implemented^13^. Moreover, the Food and Agriculture Organization of the United Nations published guidlines for water quality for agriculture^14^.

Wastewater use for irrigation in urban and peri-urban areas is mostly informal and growing in most urban cities in Africa such as Nairobi, Ghana and Dakar^15^. The primary vegetables produced are lettuce, onions and eggplant. These are preferred because they grow rapidly and profitably^16^. The major water sources are mostly from urban activities such as domestic, industrial and agriculture. Other sources include open sources of water like rivers, ponds, tanks, shallow wells, lakes, natural streams, wastewater treatment plants and house drainage spouts^17^. These sources are often polluted and below standards for irrigation purposes. For example, research done in Nairobi revealed that the wastewater used for irrigation contained heavy metals^10^. Likewise, in South Africa, vegetables produced from wastewater irrigation had traces of fecal Coliforms and heavy metals^18^.

Though the use of low-quality water is prohibited by law in some African countries such as Algeria, Tunisia and Egypt, there is no active regulation for using wastewater in vegetable irrigation^2^. Farmers and consumers of vegetables produced from wastewater irrigation have different perceptions about the practice. Informal vegetable farmers in Tanzania use wastewater cost-effective because they can’t afford potable water or treated wastewater with acceptable standards for irrigation^18^. Others prefer it because they don’t have alternative sources to irrigate during the dry season.

Three major cities in Ghana, Accra, Kumasi and Tamale, are engulfed in informal wastewater vegetable irrigation wherever there is an open surface water either polluted or not^19^. Due to poor sanitation, lack of proper drainage system and lack of wastewater treatment plant, effluent from industries and domestic sewage is often discharged into streams, rivers and open gutters^14^. It is estimated that about 47% of households discharge their liquid waste into open gutters^20^. Farmers in both urban and city peripheries make use of wastewater for vegetable irrigation. A common practice in areas of Ghana is vegetable irrigated in the dry seasons. More than 15 to 20 different kinds of vegetables are grown, all of which are sent to market. Putrefiable vegetables, including lettuce (about 95% of it in the Asante regional capital, Kumasi) are grown from urban cities. 35% is usually cultivated in Ghana's capital city, Accra. They often harvest as many as 11 times within the year^21^. Less-perishable vegetables, such as eggplants are typical of dry-season irrigation in peri-urban areas. The use of polluted water for vegetable farming is more widespread in the more populated cities where safe water is scarce and is used for domestic purposes. The general survey among farmers conducted in 2002, indicated that 84% of nearly 800 farmers farming in and close to Accra and almost all 700 farmers in Tamale used untreated wastewater for irrigation, at least during the dry seasons^22^.

According to^20^, the quality of the water use for vegetable irrigation in urban cities of Ghana is noted to be poor and below standards^23^. Antwi-Agyei reported that 160 irrigation water and 163 soil samples, 93% and 91% had levels of *E. coli*, respectively. It was found that 52% of dry season vegetable farmers in the Tamale Metropolis depended on polluted water whilst^24^. This study aimed to assess the water quality of irrigated water used by urban vegetable farmers in Tamale Metropolis, Ghana.

### Research objectives

The objectives of this paper are: (1) to investigate the quality of irrigation water used by informal peri-urban vegetable farmers in Tamale metropolis and (2) to ascertain the perceptions of farmers and consumers on irrigation water quality and vegetables produce from the practice.

### The relevance of the study

This study is timely as there has been hue and cry all over Ghana about health risk associated with the quality of the water used for urban vegetable irrigation. Many farmers are illiterates and do not know much more about the risk involved in the use of wastewater for irrigation^25^. Rising farmers awareness regarding the safe use of wastewater and improving irrigation methods will reduce related risks. This research is vital for the Tamale metropolitan assembly in enforcing its by-laws on the use of wastewater for irrigation, indiscriminate disposal of liquid waste and open defecation. It can also trigger reforms in regulatory agencies and institutions for effective monitoring of informal urban irrigation. The study will also contribute to the existing literature in academia as it focuses on analysing many chemical parameters that were not analysed before. The outcome of this paper will highlight the need for improving irrigation practices in Ghana.

## Materials and methods

First of all, we confirm that all methods including experiments, analyses and interviews were performed in accordance with relevant guidelines and regulations. These methods were approved by the Institute of Water and Energy Sciences (Including Climate Change), Pan African University (PAUWES), Tlemcen, Algeria. Moreover, we confirm that informed consent was obtained from all subjects (no subjects were under 18).

### Main irrigated sites in Tamale Metropolis

Tamale is not endowed with many water bodies unlike other cities in southern Ghana. The only water source is seasonal streams from rainfall and usually disappear during the dry season. Other sources of water include dams and dugouts^26^. The surface area is generally flat with a few valleys serving as seasonal streams. Though generally flat terrain, there are few mountains 180 m above sea level. Also, about 98 dugouts are found in communities within the metropolis used for vegetable irrigation, livestock and domestic purposes^26^. About 40% of vegetable producers are all year round with 61% of them are using water from broken sewage, septic tanks, dugouts and wells. According to WHO guidelines, these sources are unimproved accepting dugout and wells^27^. Irrigation farming is largely done in Zagyuri, Sangani and Waterworks (Gumbihene)^28^.

Zagyuri is about 8 km away from Tamale center. Farmers used untreated sewage from Kamina barracks. It covers a total of 7–12 ha according to different sources.

Sangani, 4 km from the city center, farmers used water from a dug-out meant for domestic use where the area under cultivation is between 0.5 and 4 ha.

Waterworks (Gumbihene) was originally a dam built to provide water for the metropolis. It is heavily polluted as the water flows through it from a nearby car wash station and sewage from households. The cultivated area is ranged from 13.5 to 22 ha.

### Wastewater sampling

Wastewater samples were taken once from all the sites between mid-January and late February 2018 for laboratory analysis. Sample collection bottles such as a fluoropolymer, zip-lock polyethene and ice-chest were used for both collection and transportation to the laboratory for analysis. At each study site, two samples were taken for physio-chemical analysis and one each for biological analysis.

### Farmers’ survey

Survey questions were developed and pre-tested with 45 farmers for the purpose of validity and reliability. The questionnaire was re-shaped in line with the results of the pre-testing. 45 questionnaires were administered to 15 farmers, from each site, at their farming sites in the morning and evening. The purpose of this was to unearth the sources of water used for irrigation.

### Laboratory analysis

Laboratory analyses were carried out at Water Research Institute of Council for Scientific and Industrial Research (CSIR), Tamale. Bacteriological analysis of total and faeal Coliforms, *Escherichia coli*, Total Heterotrophic Bacteria (THB) were undertaken at the Microbiological Laboratory. The physio-chemical parameters were determined using procedures stated in the Standard Methods for the Examination of Water and Wastewater^29^, pH was ascertained by pH meter. Dissolved oxygen was determined using the Wrinkler method. Nutrients (nitrates-nitrites and phosphate-) were determined using hydrazine reduction method and stannous chloride method, respectively, and heavy metals (Mn, Pb, Cu, Cd, Fe, Ar and Zn) were measured by Atomic Absorption spectrophotometry (AAS).

### Chemical analysis for heavy metals, nutrients and physio-chemical

#### Phosphate (PO4-P) by stannous chloride method

One hundred (100) ml of filtered sample was taken and 0.05 ml (1 drop) phenolphthalein indicator was added. Strong acid was added to discolourise the sample if it turned pink. 4 ml of Molybdate Reagent and 0.5 ml (10 drops) Stannous Chloride reagent was added with thorough mixing after each addition. After 10 min, but before 12 min the absorbance was measured at a wavelength of 690 nm on the Spectrophotometer- UV/VIS Ultraspec model II.

#### Nitrate–nitrogen by hydrazine reduction method

10 ml of the filtered sample was pipetted into a test tube. 1 ml of 0.3 M NaOH was added and mixed gently. 1 ml of reducing mixture which was prepared by adding 20 ml copper sulphate (CuS04) working solution and 16 ml hydrazine sulphate to 20 ml 0.3 M NaOH, was added and mixed gently. It was heated at 60 0C for 10 min in a water bath, made less hot to room temperature and 1.0 ml of colour developing reagents was added. It was shaken to mix, and the absorbance was read at 520 nm with the Spectrophotometer- UV/VIS Ultraspec model II.

#### Metals by atomic absorption spectrophotometry (AAS)

The sample was acidified to a pH of less than 2 with Nitric acid (HNO3). An instrument labelled Unilam 969 AAS with 50 mm burner was used to determine metals. A sample solution was aspirated into a flame and atomized. The metals were determined using different wavelengths, Mn(279.5 nm), Pb(283.3 nm), Cu(324.7 nm), Cd(228.8 nm), Fe(248.3 nm) and Zn(213.9 nm).

#### Measurement of total dissolve salts

Total dissolved salt represents the total concentration of salt dissolved in water. There is a relationship between the dissolved salts content of water and its electrical conductivity. The total dissolved salts values have been obtained using the conductivity meter.

#### Turbidity measurement

It is measured using nephelometer, or also known as turbidity meter. It uses light and photodetectors to measure light scatter and read out in units of nephelometric turbidity units (NTU).

### Bacteriological sampling and methods used

One water sample for bacteriological analysis was taken at each site. A plastic bottle of volume 500 ml with a plastic cap was used to collect the water samples. These bottles were sterilized before used and the mouth covered with aluminum foil to avoid contamination during sample collection. Upon collection, the samples were labelled, stored in an ice-chest at 4 °C and transported to the laboratory (WRI Laboratory, Tamale) for analyses. Membrane filtration method was used for the analysis of total coliforms, fecal coliforms, bacteria parameters. The method used conformed to the certified international procedures stated in Standard Methods for the Examination of Water and Wastewater^30^. The population of bacteria was determined using colony counter and the numbers expressed in terms of colony-forming units (CFU).

#### Membrane filtration technique

Total and fecal Coliform was determined by filtering 100 ml of the sample through a 0.45 μm filter onto a Petri dish with M—endo and MFC, respectively. They were incubated at 37 ± 2 °C and 44 ± °C, respectively for 18–24 h and the bacteria were identified, counted and recorded. Turbid water samples were serially diluted to ensure homogenous growth of bacteria on membrane filters.

#### Serial dilution preparation

A known volume, 1 ml of the actual sample was pi into a test tube with 9 ml sterile distilled water. The test tube was agitated using a vortex machine to ensure a homogenous mixture. The resultant solution became 10 ml. Another 1 ml of this product was aliquot into a second test tube with 9 ml sterile distilled water. The tube was vigorously shaken before it was replicated for another test tube. Each step of this exercise resulted in a tenfold change in the concentration from the previous concentration.

#### Dissolve oxygen by winkle method

The winkle method was used to measure dissolved oxygen in water samples collected from the three study sites. Dissolved Oxygen was used as an indicator of the health of the water body.

## Results

### Source of water for irrigation

The results of the survey showed that 64.4% of farmers used wastewater from domestic sewage, whiles 33.3% of the farmers used wastewater from the septic tank to irrigate their vegetables (Table 1). However, about 2.2% of farmers argued that water from car wash activities was also feeding into the water from domestic activities. WHO guidelines make recommendations for wastewater treatment for agriculture, suggesting waste stabilization ponds, activated sludge, trickling filtration. Water sources indicated above had no form of treatment before use by farmers. The guidelines are not adhered to due to ignorance on the part of the farmers. This calls for responsible institutions such as the National Irrigation Authority and other related organizations to develop a framework to monitor the activities of these farmers. Though related laws and policies exist, however, they lack mandate and clear regulations to regulate wastewater reuse for irrigation.

**Table 1:** Sources of irrigation water.

Water sources	Frequency	Percent
Wastewater from domestic activities	29	64.4
Septic tank	15	33.3
Other sources	1	2.2
Total	45	100.0

All the 45 farmers interviewed expressed satisfaction with all the sources due to the benefits they derive from their use. They all asserted that wastewater is available throughout the year and they do not pay for its use. Less than 10 farmers mentioned that the nutritional value of crops of the wastewater was one of the reasons they use it for irrigation. With regards to awareness on health risk associated with the use of wastewater for irrigation, 46.7% of farmers mentioned skin irritation and bad odour, they were also afraid there might be health problems due to people defecating close to water sources. About 53.3% of the farmers were of the view that there was no health risk. Below is a statement from the farmers:

“We have been in this business of wastewater irrigation for many years, and it is still the same drain water we are using. I have not heard anyone complain about this”

All the farmers were not aware of any environmental impact associated with the used of wastewater. This could be as a result of poor involvement of regulatory institutions in educating farmers on the dangers of using untreated wastewater both about the environment and human beings as well.

### Formatting of mathematical components

The results from the laboratory analysis are presented in Tables 2, 3 and 4. The mean pH values for all the sites were 6.84 and 7.79 respectively. Mean turbidity values were 19 NTU and 122 NTU whiles mean TDS range between 290.5 mg/l and 426.5 mg/l. The mean concentrations of nutrients such as ammonia, nitrate, nitrite and phosphate were recorded as 0.022 mg/l to 5.98 mg/l for ammonia, 1.06 mg/l to 7.52 mg/l for nitrate, 0.031 mg/l to 0.056 mg/l for nitrite and 0.037 mg/l to 0.069 mg/l for phosphate, respectively. Heavy metals; Mn, Fe, Cd, Ar, Cu, Zn and Pb concentrations were recorded as 0.011 mg/l to 0.275 mg/l, 0.14 mg/l to 0.54 mg/l, 0.0045 mg/l to 0.0055 mg/l, 0.001 mg/l to 0.002 mg/l, 0.02 mg/l to 0.23 mg/l, 0.005 mg/l to 0.010 mg/l and 0.0085 mg/l to 0.048 mg/l, respectively.

**Table 2:** Levels of physical parameters of wastewater/low-quality water.

Study sites	Physical parameters	Units	Value 1	Value 2	Mean
Sangani	Temperature	°C	41.2	41.8	41.5
	TDS	mg/l	289	292	290.5
	Turbidity	NTU	19	19	19
	pH	pH units	6.82	6.87	6.845
	Dissolve oxygen	mg/l	3	2.8	2.9
Kamina Barracks (Zagyuri)	Temperature	°C	28	31	29.5
	TDS	mg/l	424	429	426.5
	Turbidity	NTU	118	126	122
	pH	pH units	7.49	7.54	7.515
	Dissolve oxygen	mg/l	2.5	2.8	2.65
Waterworks (Gumbihini)	Temperature	°C	38	38.3	38.15
	TDS	mg/l	408	411	409.5
	Turbidity	NTU	120	122	121
	pH	pH units	7.8	7.79	7.795
	Dissolve oxygen	mg/l	4	4.5	4.25

**Table 3:** Mean concentration levels of nutrients in irrigation water.

Study site	Nutrients	Units	Value 1	Value 2	Mean	Max recommended concentration^14^
Sangani	Ammonia	mg/l	0.044	0.001	0.0225	0–5
	Nitrate	mg/l	6.044	9.005	7.5245	0–10
	Nitrite	mg/l	0.025	0.038	0.0315	0–10
	Phosphate	mg/l	0.074	0.025	0.0495	0–2
Kamina Barracks (Zagyuri)	Ammonia	mg/l	6.848	5.125	5.9865	0–5
	Nitrate	mg/l	0.651	1.484	1.0675	0–10
	Nitrite	mg/l	0.053	0.06	0.0565	0–10
	Phosphate	mg/l	0.029	0.046	0.0375	0–2
Waterworks (Gumbihini)	Ammonia	mg/l	0.684	0.588	0.636	0–5
	Nitrate	mg/l	2.995	2.826	2.9105	0–10
	Nitrite	mg/l	0.034	0.029	0.0315	0–10
	Phosphate	mg/l	0.083	0.056	0.0695	0–2

**Table 4:** Heavy metals mean concentrations levels.

Irrigation water source	Heavy metals	Values 1	Values 2	Mean concentrations (mg/l)	Max recommended concentration (mg/l)^14^
Sangani (dug-out)	Mn	0.043	0.072	0.0575	0.2
	Fe	0.297	0.186	0.2415	5
	Cd	0.007	0.002	0.0045	0.01
	Ar	0.001	0.003	0.002	0.1
	Cu	0.02	0.02	0.02	0.4
	Zn	0.005	0.016	0.0105	2
	Pb	0.005	0.036	0.0205	5
Kamina Baracks-Zagyuri (Wastewater from septic tank)	Mn	0.343	0.206	0.2745	0.2
	Fe	0.523	0.567	0.545	5
	Cd	0.009	0.002	0.0055	0.01
	Ar	0.001	0.001	0.001	0.1
	Cu	0.035	0.027	0.031	0.4
	Zn	0.014	0.004	0.009	2
	Pb	0.065	0.032	0.0485	5
Waterworks (Gumbihini) (Household sewerage and Car wash)	Mn	0.002	0.021	0.0115	0.2
	Fe	0.211	0.078	0.1445	5
	Cd	0.002	0.007	0.0045	0.01
	Ar	0.001	0.001	0.001	0.1
	Cu	0.45	0.011	0.2305	0.2
	Zn	0.005	0.005	0.005	2
	Pb	0.006	0.011	0.0085	5

### Physical parameters

Irrigation water from all the three sources was slightly moderate with mean TDS ranging from 290.5 to 426.5 mg/l for irrigation, which considered acceptable according to FAO water quality guidelines and the WHO guidlines of 450 mg/l to 2000 mg/l^12^. Sangani recorded the lowest TDS due to the source point of the irrigation. However, Kamina and waterworks source point were highly contaminated by domestic effluent from the military barracks close to Zagyuri community. Also, mean levels of pH of 6.84 and 7.79 were within the WHO threshold of 6.5 to 8.0^12,25^.Water samples from waterworks (Gumbihini) depicted a high level of pH due to car washing activities from a nearby washing bay using materials affect the pH levels of the stream^31^. Temperatures levels ranged between 38.15 °C and 41.5 °C, this was a result of high temperatures within the city from 25 to 45 °C during the periods from December to May. Mean turbidity of all the samples from the farming sites were 122 NTU and 19 NTU, this high above the recommended maximum concentrations with 5 NTU as a normal threshold. Sangani recorded the lowest mean TDS with 290.5NTU. The rest of the samples recorded 426.5 NTU and 409.5 NTU, respectively. From the researcher’s observation, Kamina and waterworks water samples showed high levels of TDS due to its main sources of water.

### Nutrients

Mean levels of ammonia concentrations ranged from 0.0225 to 5.985 mg/l from all three sites. These values slightley exceeded the FAO recommended levels (0.00 mg/l to 5 mg/l). Samples from Kamina irrigation sites recorded the highest concentrations of ammonia with 5.98 mg/l followed by waterworks and Sangani with concentrations of 0.636 mg/l and 0.0225 mg/l, respectively, while mean nitrate levels were slightly below the WHO limits of 10 mg/l. From the results of the study, Sangani had the highest concentration of nitrates of 7.53 mg/l seconded by Kamina and waterworks respectively. The mean level concentrations of phosphate in all the sampling points were high above the WHO recommended limit of 0.005 mg/l. Nitrite recorded lower levels of concentration from all the sample points. Moreover, nitrate and phosphate levels recorded in the study have the potential in causing eutrophication^32^, this was confirmed by the researcher’s observation at study sites such as Waterworks and Sangani. The nutrient levels of all the study sites were suitable for vegetable growth. From the field survey vegetable vendors mentioned that vegetable products from wastewater were much greener and stay fresh longer compared to vegetable products from pipe-born water. Thus, greener vegetables were considered healthy and good for consumption.

### Heavy metals concentration levels of irrigation water

Heavy metals are considered important crucial environmental pollutants and their toxicity is an issue for evolutionary, environmental, nutritional and ecological reasons. The mean heavy metals concentrations measured in all the sample sites were in the trend Ar ˃ Cd ˃ Zn ˃ Pb ˃ Cu ˃ Mn ˃ Fe (Table 4 ). The results of the study showed that mean concentrations of total iron, lead and cadmium slightly higher FAO recommended limits for vegetable irrigation. Considering the fact that about 80 per cent of vegetable products from wastewater irrigation is eaten raw^33^, lead poisoning is more likely to affect those who consume raw vegetables without the proper cleaning method. However, in reference to the level of safety, WHO concludes that there is no known level of lead exposure that is considered safe. The organization is currently developing guidelines on the prevention and management of lead poisoning. Though lead is not an important element for plants, it can contaminate soils and steadily accumulate in plants influenced by pH and particle size. Excess uptake of Pb in plants causes toxicity such as stunted growth, chlorosis as well as photosynthesis distract in plants^34^. Total irons were another excess heavy metal recorded in the study, its importance is plant growth is very crucial. Fe response to both deficiency and excess of irons by inducing different expressions. Excess of total irons in irrigation water can result in the accumulation of soils. The relatively higher concentration of heavy metals in the wastewater and well water is attributed to the influence of domestic activities from the Kamina wastewater channel as observed by^35^. Making inferences from the study, excess heavy metals in wastewater used for irrigation can further contaminate vegetables from the practice and pose health problems. Consumers in the city are more sceptical and usually ask to find out the source of vegetables before buying from vendors. Others do not care about the source but wash their vegetables before consumption. However, some are ignorant of the sources of the vegetables they consume. The bar charts below depict the mean heavy metals that exceeded the recommended limits by WHO.

### Bacteriological quality of wastewater use for vegetable irrigation

Fecal indicators microorganisms are used to determine the levels of microbiological contamination in water. *E. coli* and Total coliforms levels for Sangani, Kamina and Waterworks from the laboratory analysis were recorded as 3.2 × 10^3^ CFU 100 m/l and 5.5 × 10^2^ CFU 100 m/l, 4.0 × 10^3^ CFU 100 m/l and 1 × 10^2^ CFU 100 m/l, and 2.1 × 10^3^ CFU 100 m/l and 4.6 × 10^2^ CFU 100 m/l respectively (Table 5). Samples from Kamina recorded the highest *E. coli* and the lowest total coliforms. However, Sangani water samples recorded the highest total coliforms. The results presented in Table 5 outlines the presence of *E. coli* and Total coliforms.

**Table 5:** *E. coli* and total coliforms in irrigation water.

Sample sites	*E. coli* CFU 100 m/l	Faecal coliforms CFU/100 m/l
Sangani	3.2 × 10^3^	5.5 × 10^2^
Kamina Barracks (Zagyuri)	4.0 × 10^3^	1 × 10^2^
Waterworks (Gumbihini)	2.1 × 10^3^	4.6 × 10^2^

From the results above, all the sample points recorded higher *E. coli* and total coliforms levels more than the recommended threshold of 100–1000 CFU per 100 m/l and 10 CFU 100 m/l, respectively by WHO guidelines^12,26^. The source of the wastewater in Kamina is the main factor that influences higher levels of pathogens, the main water source that flows into the irrigation dam is from a septic tank during the dry season. Samples from Waterworks recorded the lowest *E. coli*. However, samples from Sangani though appeared protected from faecal waste showed high levels of *E. coli* and faecal coliforms compared with Waterworks samples which were more exposed to pathogen pollution by observation. Open defecation was observed taking place close to the Waterworks irrigation site. To ensure the results were accurate, three samples were taken again from Sangani to confirm its difference with the waterworks irrigation site. The activities around the dugout, as well as poor sanitation within the surrounding areas might have attributed to the presence of Coliforms in the water samples taken at Sangani irrigation site.

The presence of Coliforms in the irrigation water could contaminate vegetable products from the practice making it unwholesome for consumption. If these products are eaten without any proper cleaning methods, it could cause food poisoning. More research is needed to find out whether vegetable products from wastewater irrigation can be contaminated through practice. Moreover, the farmers were ignorant of the level of Coliform contamination. Thus, the researcher observed that they were not protected to prevent contact with the water to prevent infection^30^.

## Conclusions

Based on the results, we can conclude that total coliforms and *E. coli* in water samples were high and above the WHO recommended guidelines. This makes the water not safe for use for agriculture purposes without proper treatment. Moreover, farmers and consumers are exposed to possible contaminations. The presence of nutrients in the water samples served as a potential opportunity for food production if the WHO guidelines were to be followed. Furthermore, heavy metals concentrations were within the recommended limits of FAO with the exceptions of lead and cadmium. The practice is fueled by poor sanitation issues within the Metropolis. The water quality analysis data from this paper can be used as a baseline for the wastewater quality framework for future monitoring of irrigation in Tamale Metropolis. Through field observations, most farmers at these irrigation sites were not wearing protective materials to avoid contact with the wastewater. Therefore, safe and protective methods of farming should be adopted by farmers using wastewater to avoid contact. Moreover, according to the analysed water quality, farmers, traders and consumers need to apply further safety measures to make the vegetables safe, as outlined e.g. in^36^.
